# WDR5 facilitates recruitment of N-MYC to conserved WDR5 gene targets in neuroblastoma cell lines

**DOI:** 10.1038/s41389-023-00477-z

**Published:** 2023-06-19

**Authors:** Leigh A. Bumpous, Kylie C. Moe, Jing Wang, Logan A. Carver, Alexandria G. Williams, Alexander S. Romer, Jesse D. Scobee, Jack N. Maxwell, Cheyenne A. Jones, Dai H. Chung, William P. Tansey, Qi Liu, April M. Weissmiller

**Affiliations:** 1 Department of Biology, Middle Tennessee State University, Murfreesboro, TN 37132 USA; 2 Center for Quantitative Sciences, Vanderbilt University Medical Center, Nashville, TN 37240 USA; 3 Department of Biostatistics, Vanderbilt University Medical Center, Nashville, TN 37240 USA; 4 Department of Pediatric Surgery, University of Texas Southwestern Medical Center and Children’s Health, Dallas, TX 75234 USA; 5 Department of Biochemistry, Vanderbilt University School of Medicine, Nashville, TN 37240 USA; 6 Department of Cell and Developmental Biology, Vanderbilt University School of Medicine, Nashville, TN 37240 USA

**Keywords:** Oncogenes, Paediatric cancer

## Abstract

Collectively, the MYC family of oncoprotein transcription factors is overexpressed in more than half of all malignancies. The ability of MYC proteins to access chromatin is fundamental to their role in promoting oncogenic gene expression programs in cancer and this function depends on MYC–cofactor interactions. One such cofactor is the chromatin regulator WDR5, which in models of Burkitt lymphoma facilitates recruitment of the c-MYC protein to chromatin at genes associated with protein synthesis, allowing for tumor progression and maintenance. However, beyond Burkitt lymphoma, it is unknown whether these observations extend to other cancers or MYC family members, and whether WDR5 can be deemed as a “universal” MYC recruiter. Here, we focus on N-MYC amplified neuroblastoma to determine the extent of colocalization between N-MYC and WDR5 on chromatin while also demonstrating that like c-MYC, WDR5 can facilitate the recruitment of N-MYC to conserved WDR5-bound genes. We conclude based on this analysis that N-MYC and WDR5 colocalize invariantly across cell lines at predicted sites of facilitated recruitment associated with protein synthesis genes. Surprisingly, we also identify N-MYC-WDR5 cobound genes that are associated with DNA repair and cell cycle processes. Dissection of chromatin binding characteristics for N-MYC and WDR5 at all cobound genes reveals that sites of facilitated recruitment are inherently different than most N-MYC-WDR5 cobound sites. Our data reveals that WDR5 acts as a universal MYC recruiter at a small cohort of previously identified genes and highlights novel biological functions that may be coregulated by N-MYC and WDR5 to sustain the neuroblastoma state.

## Introduction

The *MYC* family of oncogenes encodes three related transcription factors (c-, L-, and N-MYC) that are commonly overexpressed or deregulated in diverse types of cancers [1]. The pervasive nature of MYC proteins in cancer is linked to their ability to regulate the expression of genes associated with core tumor processes [2], a function that is dependent on the heterodimerization of MYC with its obligate cofactor MAX [3, 4]. MYC-MAX heterodimers recognize and bind to E-box motifs enriched in the consensus sequence “CANNTG” that are located within promoters and enhancers of thousands of genes. Indeed, the diversity and sheer number of genes having the potential to become dysregulated upon *MYC* alteration place MYC proteins at the top of the list for targeted anti-cancer therapy pursuits.

Recently, the paradigm for how MYC-MAX recognizes its target genes has undergone reevaluation, spurred on by the identification of additional factors beyond MAX that are important for MYC to engage its target genes [5–9] and supported by mathematical modeling studies [6]. A new paradigm has been proposed called “facilitated recruitment”. In this model, selective target gene recognition by MYC is an avidity-driven process that depends on two sets of interactions: MYC-MAX heterodimers with DNA and MYC with a chromatin-resident MYC cofactor [10]. To date, only a small number of MYC cofactors have been discovered that regulate MYC target gene recognition, with the most well-studied being the chromatin regulator protein WDR5. WDR5 is best known for its role in promoting the assembly of MLL/SET methyltransferase complexes through its physical interactions within the WRAD (WDR5–RBBP5–ASH2L–DPY30) complex, which has been shown to be important in multiple cancers such as leukemia [11, 12] and glioblastoma [13]. Beyond the WRAD complex, however, WDR5 is involved in a multitude of other non-epigenetically regulated cellular processes [14]. As it relates to at least the c-MYC family member, WDR5 binds directly to a conserved region within MYC called Myc Box IIIb [5] and facilitates the recruitment of c-MYC to specific target genes involved in translational processes [9, 10], which is in line with the conserved role of WDR5 as a major regulator of protein synthesis gene expression [15]. Genetic disruption of the c-MYC–WDR5 interaction in a Burkitt lymphoma cancer context results in impaired tumorigenesis and tumor regression [9] revealing the importance of the MYC–WDR5 interaction to tumor function and implicating WDR5 as an essential MYC interaction partner [16].

Beyond Burkitt lymphoma, WDR5 is thought to play a pro-tumorigenic role in various other cancers, including high-risk neuroblastoma [15, 17], which is associated with amplification of the N-MYC gene, *MYCN* [18]. Overexpression of N-MYC is present in about 20% of total neuroblastoma cases, but up to 50% if just high-risk cases are considered [19, 20]. Moreover, increased levels of N-MYC are highly correlated with disease progression and predict poor clinical outcomes [18, 19, 21]. Although N-MYC and c-MYC are considered quite similar in terms of structure and function [18] most of what we know about N-MYC is inferred from studies on c-MYC, even though tissue and developmental specific expression differences are apparent and evidence exists that replacement of *MYCN* for *MYC* in mice is incomplete for some functions [22]. Therefore, novel and potentially impactful findings—such as the discovery of an important c-MYC cofactor like WDR5—must be investigated in the context of cancers that are maintained by N-MYC to fully understand any broad-reaching significance. Unfortunately, it is currently unknown if WDR5 can recruit N-MYC to chromatin as has been shown for c-MYC, and unclear the extent of overlap between N-MYC and WDR5 in N-MYC amplified neuroblastoma specifically. As both N- and c-MYC activate and induce genes connected to protein synthesis as part of their tumorigenic function [23–25], unraveling if WDR5 contributes to this ability would support a general paradigm for how MYC achieves this fundamental role. Furthermore, extending the MYC–WDR5 connection to additional cancers such as those maintained by N-MYC overexpression would bolster the idea that WDR5 is a context-independent MYC recruiter [10] and may allow a prediction to be made as to how targeting WDR5 in cancer would impact MYC binding to chromatin regardless of cancer context [11].

To that end, in this study we find that the ability of N-MYC to bind chromatin at known conserved WDR5-bound genes is impaired if N-MYC cannot interact with WDR5, indicating that WDR5 can facilitate recruitment of N-MYC to specific loci. Chromatin immunoprecipitation followed by next-generation sequencing (ChIP-seq) of N-MYC and WDR5 in two N-MYC amplified neuroblastoma cell lines reveals that colocalization of N-MYC and WDR5 occurs invariably at genomic regions associated with genes involved in protein synthesis and includes the sites in which WDR5 can recruit N-MYC to chromatin. Interestingly, we find that N-MYC and WDR5 also colocalize across the genome at genes seemingly unrelated to protein synthesis processes, suggesting that there are genes cobound by N-MYC and WDR5 that are regulated through a non-recruitment mechanism. Overall, our data suggest that WDR5 acts as a universal MYC recruiter at a cohort of previously identified conserved WDR5 target genes and broadens the significance of the MYC–WDR5 interaction.

## Results

### Blocking the N-MYC–WDR5 interaction impairs N-MYC binding to chromatin at conserved WDR5 target genes

**Fig. 1:**
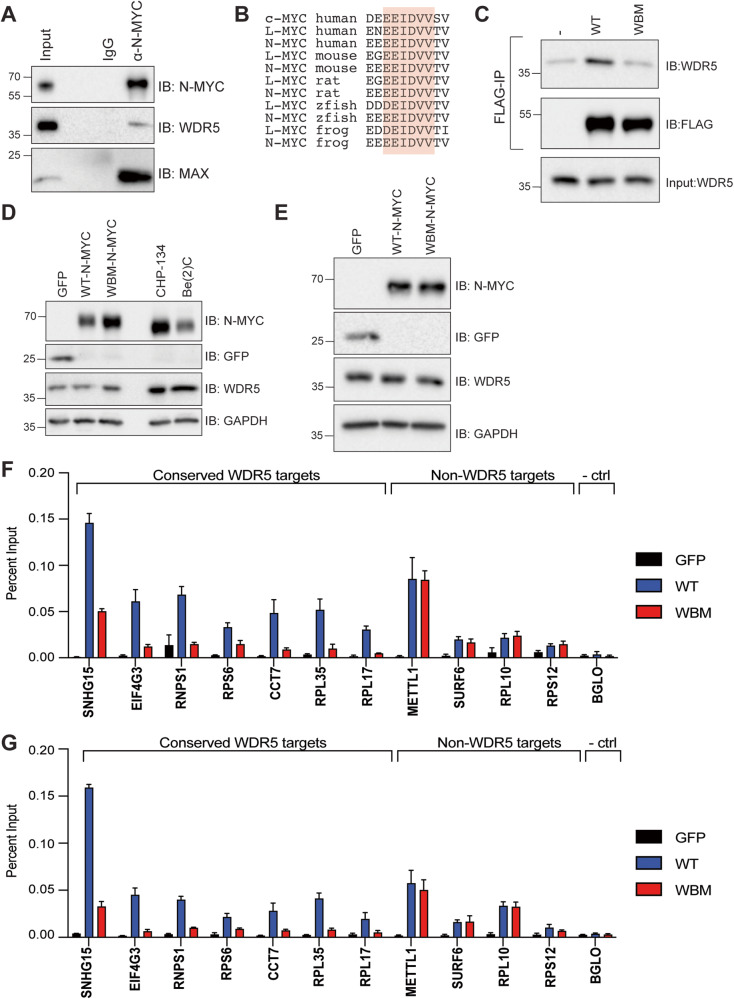
Influence of the N-MYC–WDR5 interaction on N-MYC binding to chromatin at conserved WDR5 target genes. **A** Endogenous N-MYC was immunoprecipitated from CHP-134 cells and co-immunoprecipitated material was probed for WDR5 and MAX proteins. **B** View of amino acids within Myc Box IIIb that contain the WDR5-binding motif, “IDVV”, across various organisms. **C** Flag-HA-epitope tagged Gal4 DNA binding domain alone (−) or fused to the wild-type (WT) or WBM N-MYC central portion (amino acids 133–343) were expressed in HEK293T cells and immunoprecipitation for Flag performed. Western blot analysis of co-immunoprecipitated material shows WDR5 physically interacts within the WDR5 binding motif in N-MYC. **D**, **E** SHEP (**D**) or SK-N-AS cells (**E**) were engineered to inducibly express WT-N-MYC, WBM-N-MYC, or enhanced green fluorescent protein (GFP). Western blot shows the level of expression of indicated proteins in each respective cell line following the addition of doxycycline for 24 h. GAPDH is used as a loading control. Indicated SHEP (**F**) or SK-N-AS (**G**) cell lines were induced with doxycycline for 24 h and chromatin IP (ChIP) was performed on extracted chromatin using an antibody targeted against N-MYC. The graph shows N-MYC ChIP results from induced cell lines followed by QPCR using primers targeting 7 loci that are conserved WDR5-bound sites and 4 that are not classified as WDR5-bound. *BGLO* is a negative control locus. Data represented as a percentage of input DNA for each locus (*n* = 3 biological replicates, error bars are standard error).

We first confirmed N-MYC can interact physically with WDR5 by performing immunoprecipitation (IP) for endogenous N-MYC in the N-MYC amplified neuroblastoma cell line, CHP-134. Western blots of co-immunoprecipitated material were positive for the presence of both MAX and WDR5 (Fig. 1A). To further probe the N-MYC–WDR5 interaction, we then focused on the region of MYC that interacts with WDR5. All MYC proteins contain a transcriptional activation domain and a basic helix–loop–helix DNA-binding domain separated by a stretch of roughly 150 amino acids known as the central portion. Within the central portion of all MYC family members, there are several conserved Myc Box sequences that mediate interactions of MYC with chromatin regulatory proteins [5, 9, 26–29]. WDR5 was discovered to interact directly through a short sequence of amino acids (“EEIDVV”) within Myc Box IIIb, a region that is also present within all MYC family members, including N-MYC (Fig. 1B). Expression of the central portion of N-MYC (amino acids 133–343) fused to Flag-Gal4 DNA-binding domain in HEK293T cells followed by IP for Flag confirms that WDR5 can physically interact with this region of N-MYC. (Fig. 1C). However, when three amino acids within Myc Box IIIb of N-MYC are exchanged for glutamic acids (I277E/V279E/V280E, “WBM”) [5], WDR5 is no longer co-immunoprecipitated (Fig. 1C), indicating that WDR5 physically interacts with N-MYC in a similar manner to c-MYC [5, 9]. To determine if blocking the N-MYC–WDR5 interaction impacts N-MYC chromatin binding, we engineered two neuroblastoma cell lines that lack N-MYC expression, SHEP and SK-N-AS, so that they express an inducible version of wild-type (WT) or WBM N-MYC using the Tet-on system. Both WT-, WBM-, and a control cell line that expresses enhanced green fluorescent protein (GFP) show visible protein expression within 24 h of induction **(**Fig. 1D, E). To probe the influence of the N-MYC–WDR5 interaction on the ability of N-MYC to bind chromatin, we performed ChIP coupled to a quantitative polymerase chain reaction (ChIP-QPCR) using primer sets targeted against some of the 94 genes associated with conserved and context-independent WDR5 chromatin binding sites in our previous studies [9, 15, 30, 31]. Analysis of N-MYC binding at these conserved sites shows attenuation of N-MYC binding across all sites in the WBM-samples (Fig. 1F, G, “conserved WDR5 targets”) which is selective as non-conserved sites show no overt differences in N-MYC chromatin binding. These results indicate that genetic inhibition of the N-MYC–WDR5 interaction impairs N-MYC binding to chromatin at specific sites across the genome that are well-documented and conserved WDR5 targets.

### Acute depletion of WDR5 attenuates N-MYC binding to chromatin in a N-MYC-amplified neuroblastoma cell line

**Fig. 2:**
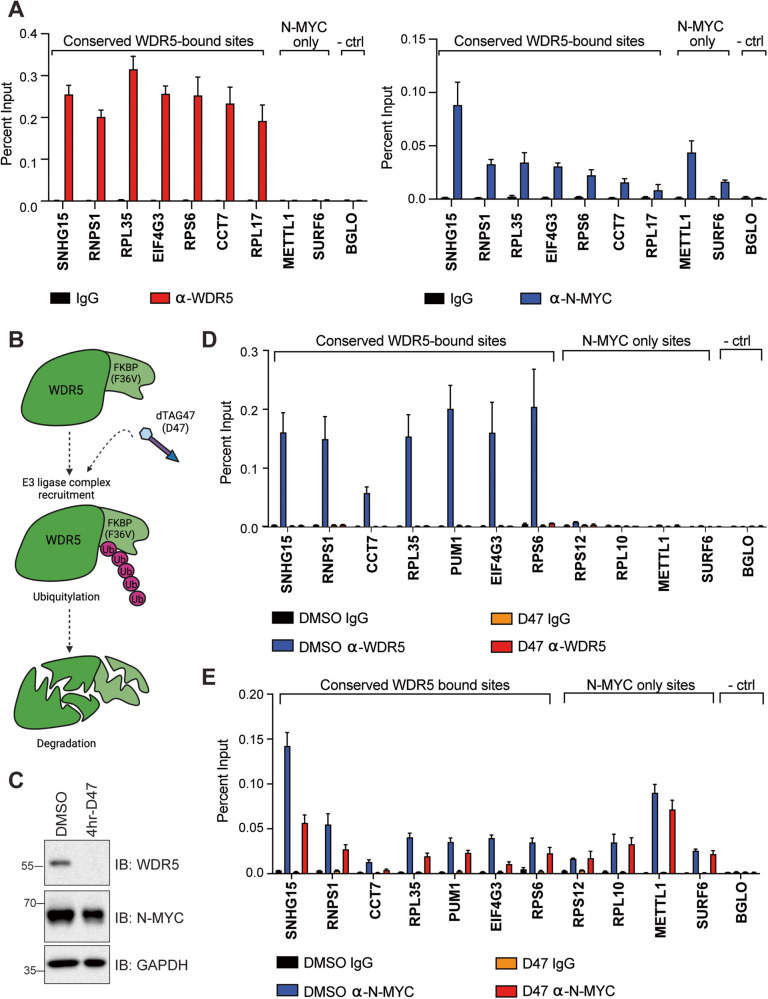
Impact of WDR5 depletion on the ability of N-MYC to bind chromatin in N-MYC amplified neuroblastoma cells. **A** Co-immunoprecipitated DNA from ChIP for N-MYC and WDR5 in IMR-32 cells was probed by QPCR using primers targeting 7 loci that are conserved WDR5-bound sites and 2 that are not classified as WDR5-bound. *BGLO* is a negative control locus. Data represented as a percentage of input DNA for each locus (*n* = 3 biological replicates, error bars are standard error). **B** CHP-134 cells were engineered previously to express a version of WDR5 fused to the FKBP(F36V) degron [15] (“called DTWDR5 cells”). In these cells, the addition of dTAG47 (D47) links WDR5 to an E3 ligase complex, resulting in ubiquitylation and subsequent rapid degradation through the proteosome system. Image created with BioRender.com. **C** Western blot showing the level of WDR5 expression in DTWDR5 cells following 4 h treatment with 500 nM D47 compared to matched DMSO control. GAPDH is used to control loading. **D** DTWDR5 cells were treated as in (**C**) and ChIP was performed for WDR5 following extraction of chromatin. Graph shows that chromatin-bound WDR5 is absent upon D47 treatment at 7 loci that are conserved WDR5-bound sites. **E** DTWDR5 cells were treated as in (**C**) and ChIP was performed for N-MYC. At sites where WDR5 is absent (**D**), N-MYC binding is reduced. For both (**D**) and (**E**), *BGLO* is a negative control locus. Data represented as a percentage of input DNA for each locus (*n* = 3 biological replicates, error bars are standard error).

To begin to dissect the interplay between N-MYC and WDR5 we performed ChIP-QPCR in the N-MYC amplified cell line, IMR-32, using antibodies targeted against N-MYC or WDR5. At conserved WDR5-bound sites, N-MYC and WDR5 co-localize as expected (Fig. 2A). To assess if WDR5 can impact the recruitment of N-MYC to chromatin as seen in the SHEP/SK-N-AS cell systems, we used CHP-134 cells that we previously engineered to express a version of WDR5 that can be acutely depleted by the dTAG approach (Fig. 2B) [15, 32]. Upon addition of a dTAG47 molecule for 4 h, WDR5 is depleted from the cells, consistent with past observations [15] (Fig. 2C). ChIP-QPCR analysis at this same time point reveals that chromatin-bound WDR5 is also absent (Fig. 2D) and that N-MYC binding is reduced at sites in which WDR5 is lost (Fig. 2E), although the level of reduction using the dTAG system is not as striking as that seen when WBM-N-MYC was assessed (Fig. 1F, G). Again, the effect on N-MYC chromatin binding shows a bias for sites in which WDR5 is bound to chromatin as non-WDR5-bound sites retain N-MYC binding (Fig. 2E).

### N-MYC and WDR5 colocalize extensively in the CHP-134 neuroblastoma cell line

**Fig. 3:**
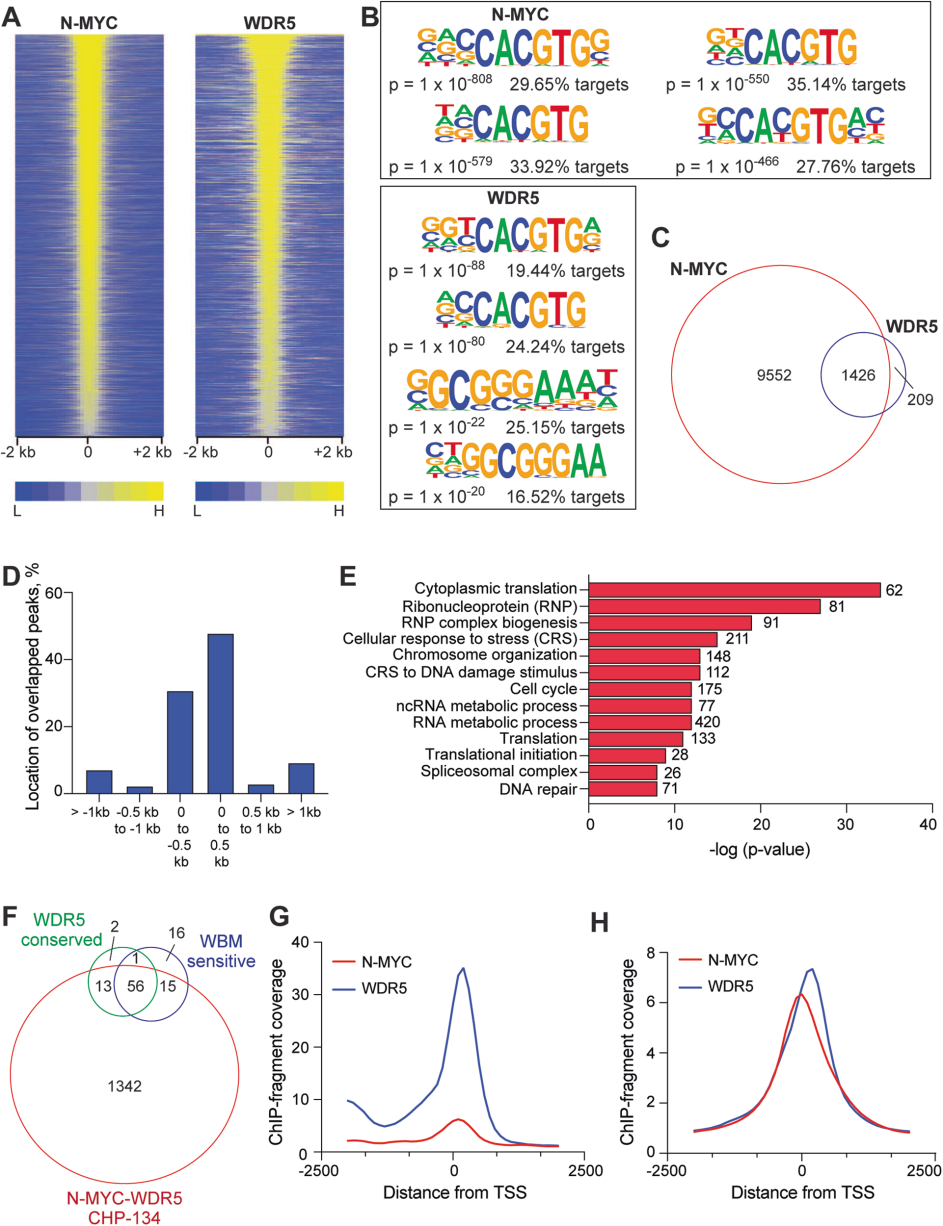
Colocalization of N-MYC and WDR5 on chromatin in CHP-134 cells. **A** Heatmap showing normalized ChIP-seq peaks intensity for N-MYC and WDR5 detected genome-wide in CHP-134 cells. Heatmaps show signal in 100 bp bins within 2 kb from the center of peaks. **B** Examples from known motif enrichment analysis for N-MYC and WDR5 peaks. “CACGTG” is a canonical E-box motif. **C** Venn diagram showing the overlap between total peaks detected for N-MYC and WDR5. Overlap was called if individual peaks fall within 1 bp of each other. **D** Distribution of N-MYC–WDR5 cobound sites based on the annotation of overlapped peaks to their nearest TSS. **E** Gene ontology enrichment analysis on genes that show N-MYC–WDR5 colocalization within 1 kb of their TSS. The significance of enrichment is located on the *x*-axis and the numbers next to the red bars are the number of genes in each category. **F** Venn diagram showing the overlap of N-MYC–WDR5 cobound binding sites with the conserved WDR5 binding sites across human cell lines [15] or c-MYC binding sites that are WBM-sensitive [9]. **G** Average normalized ChIP-seq fragment coverage for N-MYC (red) and WDR5 (blue) at the 94 conserved WDR5-target genes. **H** Average normalized ChIP-seq fragment coverage for N-MYC (red) and WDR5 (blue) at all N-MYC–WDR5 cobound sites detected.

To gain insight into the degree of N-MYC and WDR5 colocalization beyond conserved WDR5-bound sites that we can track by QPCR, we performed ChIP-seq for N-MYC and WDR5 in CHP-134 cells. We detected ~1600 peaks for WDR5 and 10,000 peaks for N-MYC (Fig. 3A, C) each of which was enriched with E-box motifs (“CACGTG”, Fig. 3B). N-MYC peaks alone were localized almost equally at transcription start site (TSS) proximal and distal sites (Supplementary Fig. 1A), but the majority (~80%) of WDR5 peaks were called within 1 kb of a TSS (Supplementary Fig. 1B). Strikingly, 87% of WDR5 peaks detected overlap with N-MYC peaks (Fig. 3C, Supplementary Table 1), revealing that most of WDR5 detected in CHP-134 cells colocalizes with N-MYC. Consistent with the location of WDR5 alone, over 80% of N-MYC–WDR5 overlapped sites occur within 1 kb of the nearest TSS (Supplementary Fig. 1C). Detailed analysis of N-MYC–WDR5 overlapped peaks reveals that most overlapping peaks are centered within 500 bp upstream and downstream of the associated TSS (Fig. 3D, Supplementary Fig. 1D), which is a characteristic of chromatin-bound WDR5 observed across multiple human and mouse-derived cell lines [15]. Annotation of N-MYC–WDR5 overlapped peaks to their nearest gene and subsequent gene ontology (GO) term analysis (https://david.ncifcrf.gov/) shows cobound sites are associated with genes involved in various important biological functions such as RNA processing, ribosomes, and translation all of which are known conserved WDR5-related functions [9, 15, 30, 31]. Interestingly, unique gene categories not previously identified as conserved WDR5 genes are cobound by N-MYC and WDR5 including genes involved in chromosome organization, DNA repair, cell cycle, and spliceosome functions (Fig. 3E).

Facilitated recruitment of c-MYC to chromatin by WDR5 occurs at <100 WBM-sensitive genes [9] predominantly associated with protein synthesis processes and includes many of the conserved WDR5-bound genes we focused our initial ChIP-QPCR analysis on (Fig. 1, Fig. 2). To provide a direct comparison between all datasets, we compared N-MYC–WDR5 binding sites to the context-independent WDR5 chromatin binding sites across human cell lines [15] or c-MYC binding sites called as WBM-sensitive [9]. In doing this analysis, we see that ~81% of the proposed sites in which WDR5 recruits c-MYC are also N-MYC–WDR5 cobound in CHP-134 cells and nearly all the conserved WDR5 binding sites also show N-MYC binding (Fig. 3F). This suggests that N-MYC is present at conserved WDR5 binding sites and that WDR5 recruits N-MYC to chromatin at a small cohort of genes connected to protein synthesis. To determine if conserved sites show any differences in binding characteristics that could indicate this unique mode of regulation, we extracted the average ChIP-seq profiles for N-MYC and WDR5 at the conserved WDR5-bound genes and compared this to the average ChIP-seq profiles at all N-MYC–WDR5 cobound sites. Two apparent differences are observed: (1) the intensity of WDR5 is ~3 times higher on average at the conserved genes (Fig. 3G, H, Supplementary Fig. 1E) and (2) the average N-MYC ChIP-seq signal is shifted downstream of the TSS at conserved genes, aligning directly under the WDR5 peak. Together this suggests that sites of facilitated recruitment are inherently different than all other N-MYC–WDR5 cobound sites. Furthermore, by extension it also implies that the newly identified gene categories that we find associated with N-MYC–WDR5 colocalization are not sites in which WDR5 recruits N-MYC to chromatin but instead may be genes coregulated by N-MYC and WDR5, highlighting a novel biological interplay that has yet to be explored. In sum, we conclude that in CHP-134 cells N-MYC and WDR5 colocalize at predicted sites of facilitated recruitment [9, 15], which is consistent with our findings that WDR5 can influence the ability of N-MYC to bind at select genes (Figs. 1F, G and 2E).

### Invariant N-MYC–WDR5 cobound sites occur at sites linked to genes involved in protein synthesis

**Fig. 4:**
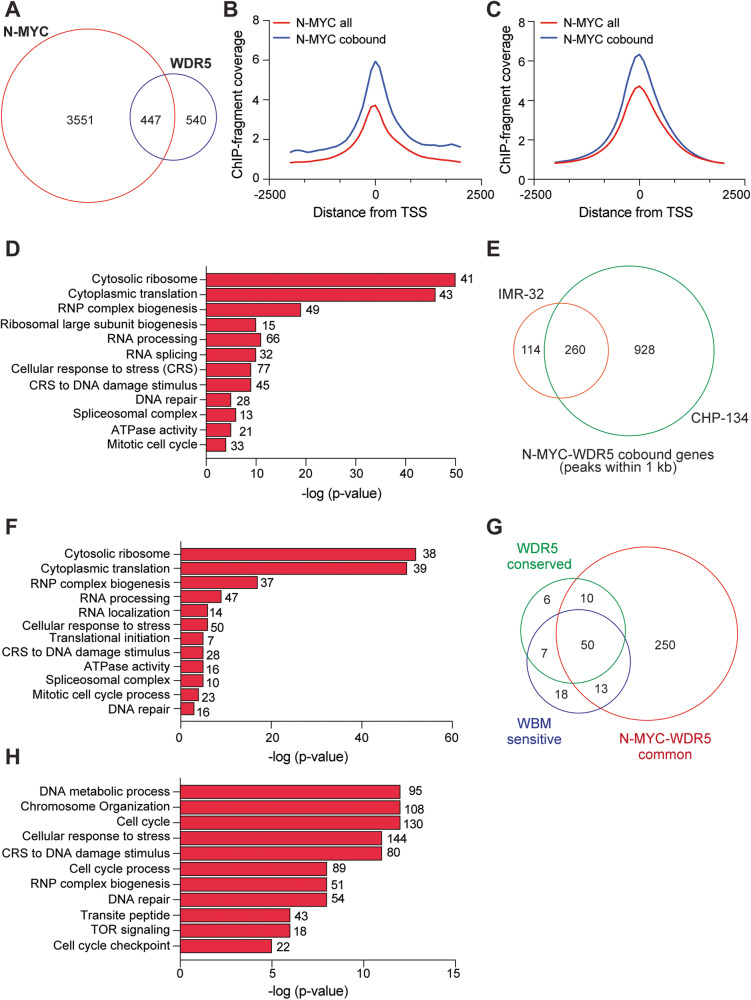
Colocalization of N-MYC and WDR5 on chromatin in IMR-32 cells is similar to CHP-134 cells. **A** Venn diagram showing the overlap between total peaks detected for N-MYC and WDR5 in IMR-32 cells. Overlap was called if individual peaks fall within 1 bp of each other. **B** Average normalized ChIP-seq fragment coverage for N-MYC at all sites centered at the TSS (red) compared to N-MYC at N-MYC–WDR5 cobound sites (blue) in IMR-32 cells. **C** Average normalized ChIP-seq fragment coverage for N-MYC at all sites centered at the TSS (red) compared to N-MYC at N-MYC–WDR5 cobound sites (blue) in CHP-134 cells. **D** Gene ontology enrichment analysis of genes that show N-MYC–WDR5 colocalization within 1 kb of their TSS in IMR-32 cells. The significance of enrichment is located on the *x*-axis and the numbers next to the red bars are the number of genes in each category. **E** Venn diagram showing the overlap between N-MYC–WDR5 cobound genes in each cell line. Only genes showing N-MYC–WDR5 bound within 1 kb of the gene were included. **F** Gene ontology enrichment analysis of 260 common genes from (**E**). Significance of enrichment is located on the *x*-axis and the numbers next to the red bars are the number of genes in each category. **G** Venn diagram showing the overlap of common N-MYC–WDR5 cobound sites with the conserved WDR5 binding sites across human cell lines [15] or c-MYC binding sites that are WBM-sensitive [9]. **H** Gene ontology enrichment analysis of 928 N-MYC-WDR5 cobound genes from (**E**) that are specific to CHP-134 cells. Significance of enrichment is located on the *x*-axis and the numbers next to the red bars are the number of genes in each category. RNP ribonucleoprotein.

As a second point of comparison for understanding how N-MYC and WDR5 feature in N-MYC-amplified cell lines, we performed ChIP-seq for N-MYC and WDR5 in IMR-32 cells. Overall, we detected ~990 peaks for WDR5 and 4000 peaks for N-MYC in this cell line. ~63% of detected WDR5 peaks and almost all of the peaks detected for N-MYC were also peaks called in CHP-134 cells (Supplementary Fig. 2A, B). While the number of N-MYC peaks detected is lower in IMR-32 cells and does not correlate with steady-state protein levels (Supplementary Fig. 1F), the nature of chromatin binding is similar to CHP-134 cells as N-MYC peaks alone show a mixture of TSS-proximal and distal binding (Supplementary Fig. 2C), while WDR5 peaks are proximally localized (Supplementary Fig. 2D). In contrast to CHP-134 cells, only ~45% of WDR5 peaks overlap with N-MYC peaks (Fig. 4A), indicating that the extent of colocalization between N-MYC and WDR5 can be variable across N-MYC amplified cell lines. However, cobound sites preserve several features noted in CHP-134 cells such as predominant TSS-proximal binding (Supplementary Fig. 2E–G). We also observe that the intensity of N-MYC is higher when N-MYC colocalizes with WDR5 (Fig. 4B), a phenomenon that is true in CHP-134 cells as well (Fig. 4C). Furthermore, annotation of N-MYC–WDR5 overlapped peaks to their nearest gene (Supplementary Table 2) and subsequent GO term analysis shows cobound sites in IMR-32 cells are associated with genes heavily enriched within biological functions related to translation and ribosomes (Fig. 4D), consistent with cobound genes identified in CHP-134 cells. In addition, newly identified gene categories such as those pertaining to DNA repair, cell cycle, and the spliceosome complex are also associated with N-MYC–WDR5 cobound sites.

Direct comparison of N-MYC–WDR5 cobound genes across both CHP-134 and IMR-32 cells reveals nearly 70% of genes that are N-MYC–WDR5 cobound in IMR-32 cells are also genes cobound in CHP-134 cells (Fig. 4E), suggesting the existence of a common set of genes consistently bound by both N-MYC and WDR5. Not surprisingly, the commonly shared genes are significantly enriched in functional categories related to translation and protein synthesis (Fig. 4F). A separate comparison of overlapping N-MYC–WDR5 binding sites with conserved WDR5 binding sites across human cells or WBM-sensitive c-MYC binding sites shows that the N-MYC–WDR5 cobound sites found in both CHP-134 and IMR-32 overlap with nearly all these separate datasets (Fig. 4G), indicating that predicted sites of facilitated recruitment can be identified across multiple cell lines. Unique N-MYC–WDR5 cobound sites that are associated with genes linked to DNA repair and cell cycle functions are also common in both cell lines, suggesting that N-MYC and WDR5 may coregulate unique genes not previously appreciated as conserved WDR5 targets due to context-specificity. Analysis of the 928 N-MYC–WDR5 cobound genes unique to CHP-134 (Fig. 4E) reemphasize that these types of genes may be context-specific as GO term analysis reveals that the CHP-134-specific cobound genes remain enriched in functions such as DNA repair and cell cycle processes (Fig. 4H). Taken together, our results reveal that across multiple N-MYC-amplified neuroblastoma cell lines, N-MYC and WDR5 colocalize invariably at genes linked to protein synthesis, but that context-dependent N-MYC–WDR5 cobound genes may be an important factor to keep in mind when considering how N-MYC and WDR5 ultimately influence N-MYC-amplified neuroblastoma.

## Discussion

The ability of MYC proteins to bind chromatin at the genes they regulate is key to their function as transcription factors. The accepted paradigm for how this is achieved is that MYC heterodimerizes with MAX, which together binds to specific sequences located throughout the genome. New evidence continues to be uncovered that supports the notion that MYC–MAX interactions alone cannot explain all MYC binding to chromatin [6] and it is clear that further study is warranted to fully understand how MYC proteins select their targets. In this study, we investigated the interaction of N-MYC with WDR5, a conserved chromatin-bound regulator of protein synthesis gene expression [15] and a cofactor that c-MYC uses for specific target gene selection [5, 9]. While certainly, the number of c-MYC–WDR5 cobound genes identified previously was small in comparison to the number of total c-MYC-targets identified [9], they contained numerous tumor-critical genes encoding ribosomal protein subunits, nucleolar RNAs, and translation initiation factors, all of which when disconnected from the totality of c-MYC gene expression was enough to result in tumor failure in a Burkitt lymphoma model [9]. Therefore, knowing how much of this can be extended to N-MYC and N-MYC-amplified neuroblastoma is beneficial in determining the broad significance of WDR5 as a context-independent MYC cofactor.

We find in the present work that genetic inhibition of the interaction of N-MYC with WDR5 blocks the ability of N-MYC to bind chromatin at known conserved WDR5 targets (Fig. 1). In N-MYC-amplified neuroblastoma cell lines, removal of WDR5 from cells also attenuates the ability of N-MYC to bind these same sites (Fig. 2), indicating that WDR5 can recruit N-MYC to chromatin at specific regions of the genome as has been seen for c-MYC. Genome-wide chromatin binding assays in multiple cell lines showed that depending on the cell line, ~45–85% of WDR5 detected colocalizes on chromatin with N-MYC (Figs. 3C and 4A), and that cobound sites are associated with genes linked to protein synthesis function such as those encoding ribosomal proteins, translation initiation factors, and RNA processing factors (Fig. 4F). Many of these are predicted to be sites of facilitated recruitment based on their extensive overlap with conserved WDR5-bound sites and WBM-sensitive c-MYC-binding sites (Figs. 3F and 4G). Overall, these results make a strong case for WDR5 being able to influence the ability of N-MYC to bind chromatin at a small group of genes involved in maintaining the cell proteome and are consistent with previous studies focused either on c-MYC or WDR5 alone.

Interestingly, in both cell lines N-MYC and WDR5 also colocalize at non-protein synthesis genes, including DNA repair and damage genes, cell cycle genes, and spliceosome complex genes (Figs. 3E and 4D, F). However, it is unlikely that most of these newly identified N-MYC–WDR5 cobound sites are regulated by a direct recruitment mechanism based on what is known about conserved WDR5-bound sites and WBM-sensitive c-MYC binding sites. In support of this, extraction of the chromatin binding properties for the 94 conserved WDR5-bound genes show these predicted sites of facilitated recruitment are inherently different than all other N-MYC–WDR5 cobound sites in that N-MYC and WDR5 peaks are more closely aligned with each other and the intensity of WDR5 is typically higher at these regions (Fig. 3F–H). Determination of a common set of N-MYC–WDR5 cobound sites across CHP-134 and IMR-32 cell lines reveals that most of these common binding sites overlap with the conserved set of WDR5-bound sites (Fig. 4G), suggesting that N-MYC—or any other MYC family member for that matter—is most likely found with WDR5 at these regions regardless of cancer context.

Previous in-depth analysis of WDR5 chromatin binding across diverse mouse and human cell lines showed that the number of WDR5 binding sites across cell lines can vary by 10-fold, depending on the context, which was not due to differences in ChIP efficiency [15]. In CHP-134 and IMR-32 cell lines, we do not see that degree of variation as in each cell line ~1000 WDR5 peaks are detected (Figs. 3C and 4A). However, if WDR5 ChIP-seq data in the N-MYC-amplified Be(2)C cell line is considered there does exist some variation within N-MYC-amplified neuroblastoma cell lines as only ~250 WDR5 peaks were detected in Be(2)C cells, the majority of which included the conserved set of WDR5 targets [15]. The reason for this may be due to the heterogenous nature of neuroblastoma cells [33, 34] of which CHP-134 and IMR-32 cells grown in a culture mostly consist of neuroblasts (classified as “N-type”) while Be(2)C cells show an intermediate phenotype (classified as “I-type”). Regardless, because WDR5 detected in Be(2)C cells is consistent with conserved, context-independent binding of WDR5, we predict based on our data that N-MYC–WDR5 colocalization and facilitated recruitment would be present in the Be(2)C cell line as well.

Currently, it is unknown if and how N-MYC and WDR5 coregulate the unique N-MYC–WDR5 cobound genes identified in these cell lines (Fig. 4F, H). It is also unclear how context-dependent differences in N-MYC–WDR5 colocalization influence the N-MYC-mediated transcriptome. The differences in which genes are cobound by N-MYC and WDR5 may need to be considered when thinking about the outcome of targeting WDR5 with small molecules—an approach that has gained attention over the past several years [35–38]—and most recently has been shown to be effective as a potential therapy for disrupting cancer stem cell function in glioblastoma [13]. N-MYC is overexpressed in subsets of glioblastoma [39] and therefore some of the findings in this study may be applicable to understanding the totality of how WDR5 inhibition impacts glioblastoma growth and function. In addition, as N-MYC is also overexpressed or amplified in medulloblastoma, retinoblastoma, and subsets of breast, prostate, and small-cell lung cancers [21], the work presented here may have implications for more than just high-risk neuroblastoma in general.

## Materials and methods

Information pertaining to cell culture and cell line engineering, plasmid generation, protein lysate and immunoprecipitations, and Western blot can be found within Supplementary Material.

### ChIP-QPCR and ChIP-seq

Approximately 7.5–10 × 10^6^ SHEP or SK-N-AS cells induced with 1 µg/ml doxycycline for 24 h were harvested for each chromatin immunoprecipitation (ChIP) sample. To crosslink DNA–protein complexes, 1% formaldehyde was added to each plate for 10 min, followed by the addition of 0.125 M glycine to quench the reaction. Cells were washed twice with ice-cold PBS and collected from the plate. Fixed cells were pelleted by centrifugation and nuclei were extracted by incubating the cells for 5 min on ice in a nuclear lysis buffer (10 mM HEPES, pH 7.9, 10 mM KCl, 0.4% NP-40) supplemented with PMSF and protease inhibitor cocktail (Roche). Nuclei were pelleted at 1500 RPM and lysed for 15 min in FALB buffer (50 mM HEPES, pH 7.5, 1% Triton, 1 mM EDTA, 140 mM NaCl) containing 1% SDS, PMSF, and protease inhibitor cocktail (Roche). Chromatin was sheared using a Diagenode Bioruptor Plus instrument and then debris was clarified through centrifugation. Each chromatin sample was stored at −80 °C until ChIP was performed. For DTWDR5 CHP-134 cells, chromatin was collected in the same manner following a 4 h treatment with 500 nM dTAG47 or DMSO-matched control. For ChIP-QPCR, chromatin from each sample was diluted in ice-cold FALB supplemented with PMSF and protease inhibitor cocktail (Roche) but containing no SDS. Immunoprecipitation was performed with 5 µl of an antibody against N-MYC (Cell Signaling, 51705), WDR5 (Cell Signaling, 13105), or 800 ng of normal rabbit IgG control (Cell Signaling, 2729) overnight at 4 °C. The following day, protein A agarose (Fisher Scientific) was blocked for 30 min with 1% BSA in FALB containing no SDS and allowed to bind immunocomplexes for 2–4 h at 4 °C. All ChIP samples were washed extensively as previously described [40, 41] and then ChIP and input samples decrosslinked at 65 °C overnight in 1X TE (10 mM Tris, pH 8.0, 1 mM EDTA) containing 0.1% SDS and 20 µg Proteinase K. ChIP-QPCR samples were diluted in 1X TE and co-immunoprecipitated DNA analyzed on an AriaMX QPCR machine using Perfecta SYBR Green FastMix (QuantaBio) and primers listed in Supplementary Table 3. For ChIP-seq, chromatin from 10 × 10^6^ IMR-32 cells or CHP-134 cells treated with 0.005% dimethyl sulfoxide was extracted as described above. ChIP was performed using 5 µl of anti-WDR5 (Cell Signaling, 13105), 800 ng of normal rabbit IgG control (Cell Signaling, 2729S), or 5 µl of custom serum to target N-MYC [42], which was provided as a gift from Dr. Huck-Hui Ng. All ChIP-QPCR and ChIP-seq experiments were performed three independent times as noted in the figure legends. ChIP-seq samples were processed identically to ChIP-QPCR samples except following decrosslinking three ChIPs from identical samples were pooled together and purified using a Qiagen PCR purification kit. Eluted DNA was used to generate libraries as previously described [40] using the Ultra II DNA Library Prep protocol (New England Biolabs). Sequencing data for CHP-134 and IMR-32 samples were obtained on an Illumina NextSeq500 with 75 bp single reads and an Illumina NovaSeq 6000 with 150 bp paired end reads, respectively. All sequencing was completed by the Vanderbilt Technologies for Advanced Genomics Core located at Vanderbilt University Medical Center.

### ChIP-Seq analysis

After adapter removal via Cutadapt [43], alignment of the sequencing reads to the Hg19 human genome was performed using Bowtie2 [44]. To call peaks, MACS2 was used with a *q*-value of 0.05 [45]. DiffBind [46] was then used to identify consensus peaks for each sample with peaks present in at least two replicates being included. Mapped tags were normalized to 10 million to generate the histograms of the ChIP-seq profile. Cobound sites for N-MYC and WDR5 were called if their individual peaks overlapped by at least one base pair. Annotation of peaks and motif analysis was generated using Homer annotatePeaks command line with default settings (http://homer.ucsd.edu/homer/) and Homer: findMotfisGenome. GO term enrichment analysis was performed using functional annotation clustering through DAVID (https://david.ncifcrf.gov/).

## Supplementary information

Supplementary Material

Supplementary Table 1

Supplementary Table 2

Supplementary Table 3

## Data Availability

ChIP-seq datasets generated in this study are deposited in GEO under GSE222157. Additional data is provided upon request.

## References

[1] Jones CA, Tansey WP, Weissmiller AM (2022). Emerging themes in mechanisms of tumorigenesis by SWI/SNF subunit mutation. Epigenet Insights.

[2] Tansey WP (2014). Mammalian MYC proteins and cancer. N J Sci.

[3] Blackwood EM, Eisenman RN (1991). Max: a helix–loop–helix zipper protein that forms a sequence-specific DNA-binding complex with Myc. Science.

[4] Jones S (2004). An overview of the basic helix–loop–helix proteins. Genome Biol.

[5] Thomas LR, Wang Q, Grieb BC, Phan J, Foshage AM, Sun Q (2015). Interaction with WDR5 promotes target gene recognition and tumorigenesis by MYC. Mol Cell.

[6] Lorenzin F, Benary U, Baluapuri A, Walz S, Jung LA, von Eyss B (2016). Different promoter affinities account for specificity in MYC-dependent gene regulation. eLife.

[7] Richart L, Carrillo-de Santa Pau E, Rio-Machin A, de Andres MP, Cigudosa JC, Lobo VJ (2016). BPTF is required for c-MYC transcriptional activity and in vivo tumorigenesis. Nat Commun.

[8] Gerlach JM, Furrer M, Gallant M, Birkel D, Baluapuri A, Wolf E (2017). PAF1 complex component Leo1 helps recruit Drosophila Myc to promoters. Proc Natl Acad Sci USA.

[9] Thomas LR, Adams CM, Wang J, Weissmiller AM, Creighton J, Lorey SL (2019). Interaction of the oncoprotein transcription factor MYC with its chromatin cofactor WDR5 is essential for tumor maintenance. Proc Natl Acad Sci USA.

[10] Thomas LR, Foshage AM, Weissmiller AM, Tansey WP (2015). The MYC–WDR5 nexus and cancer. Cancer Res.

[11] Aho ER, Weissmiller AM, Fesik SW, Tansey WP (2019). Targeting WDR5: a WINning anti-cancer strategy?. Epigenet Insights.

[12] Liu L, Guo X, Wang Y, Li G, Yu Y, Song Y (2023). Loss of Wdr5 attenuates MLL-rearranged leukemogenesis by suppressing Myc targets. Biochim Biophys Acta Mol Basis Dis.

[13] Mitchell K, Sprowls SA, Arora S, Shakya S, Silver DJ, Goins CM (2023). WDR5 represents a therapeutically exploitable target for cancer stem cells in glioblastoma. Genes Dev.

[14] Guarnaccia AD, Tansey WP (2018). Moonlighting with WDR5: a cellular multitasker. J Clin Med.

[15] Bryan AF, Wang J, Howard GC, Guarnaccia AD, Woodley CM, Aho ER (2020). WDR5 is a conserved regulator of protein synthesis gene expression. Nucleic Acids Res.

[16] Thomas LR, Adams CM, Fesik SW, Eischen CM, Tansey WP (2020). Targeting MYC through WDR5. Mol Cell Oncol.

[17] Sun Y, Bell JL, Carter DR, Gherardi S, Poulos RC, Milazzo G (2015). WDR5 supports an N-Myc transcriptional complex that drives a pro-tumorigenic gene expression signature in neuroblastoma. Cancer Res.

[18] Huang M, Weiss WA (2013). Neuroblastoma and MYCN. Cold Spring Harb Perspect Med.

[19] Castel V, Grau E, Noguera R, Martinez F (2007). Molecular biology of neuroblastoma. Clin Transl Oncol.

[20] Rickman DS, Schulte JH, Eilers M (2018). The expanding world of N-MYC-driven tumors. Cancer Discov.

[21] Beltran H (2014). The N-myc oncogene: maximizing its targets, regulation, and therapeutic potential. Mol Cancer Res.

[22] Malynn BA, de Alboran IM, O’Hagan RC, Bronson R, Davidson L, DePinho RA (2000). N-myc can functionally replace c-myc in murine development, cellular growth, and differentiation. Genes Dev.

[23] Kim S, Li Q, Dang CV, Lee LA (2000). Induction of ribosomal genes and hepatocyte hypertrophy by adenovirus-mediated expression of c-Myc in vivo. Proc Natl Acad Sci USA.

[24] Boon K, Caron HN, van Asperen R, Valentijn L, Hermus MC, van Sluis P (2001). N-myc enhances the expression of a large set of genes functioning in ribosome biogenesis and protein synthesis. EMBO J.

[25] Dang CV (2013). MYC, metabolism, cell growth, and tumorigenesis. Cold Spring Harbor Perspect Med.

[26] Kurland JF, Tansey WP (2008). Myc-mediated transcriptional repression by recruitment of histone deacetylase. Cancer Res.

[27] Thomas LR, Foshage AM, Weissmiller AM, Popay TM, Grieb BC, Qualls SJ (2016). Interaction of MYC with host cell factor-1 is mediated by the evolutionarily conserved Myc box IV motif. Oncogene.

[28] Popay TM, Wang J, Adams CM, Howard GC, Codreanu SG, Sherrod SD (2021). MYC regulates ribosome biogenesis and mitochondrial gene expression programs through its interaction with host cell factor-1. eLife.

[29] Cowling VH, Chandriani S, Whitfield ML, Cole MD (2006). A conserved Myc protein domain, MBIV, regulates DNA binding, apoptosis, transformation, and G2 arrest. Mol Cell Biol.

[30] Aho ER, Wang J, Gogliotti RD, Howard GC, Phan J, Acharya P (2019). Displacement of WDR5 from chromatin by a WIN site inhibitor with picomolar affinity. Cell Rep.

[31] Florian AC, Woodley CM, Wang J, Grieb BC, Slota MJ, Guerrazzi K (2022). Synergistic action of WDR5 and HDM2 inhibitors in SMARCB1-deficient cancer cells. NAR Cancer.

[32] Nabet B, Roberts JM, Buckley DL, Paulk J, Dastjerdi S, Yang A (2018). The dTAG system for immediate and target-specific protein degradation. Nat Chem Biol.

[33] Shimada H, Chatten J, Newton WA, Sachs N, Hamoudi AB, Chiba T (1984). Histopathologic prognostic factors in neuroblastic tumors: definition of subtypes of ganglioneuroblastoma and an age-linked classification of neuroblastomas. J Natl Cancer Inst.

[34] Walton JD, Kattan DR, Thomas SK, Spengler BA, Guo HF, Biedler JL (2004). Characteristics of stem cells from human neuroblastoma cell lines and in tumors. Neoplasia.

[35] Han QL, Zhang XL, Ren PX, Mei LH, Lin WH, Wang L (2023). Discovery, evaluation and mechanism study of WDR5-targeted small molecular inhibitors for neuroblastoma. Acta Pharmacol Sin.

[36] Guarnaccia AD, Rose KL, Wang J, Zhao B, Popay TM, Wang CE (2021). Impact of WIN site inhibitor on the WDR5 interactome. Cell Rep.

[37] Tian J, Teuscher KB, Aho ER, Alvarado JR, Mills JJ, Meyers KM (2020). Discovery and structure-based optimization of potent and selective WD repeat domain 5 (WDR5) inhibitors containing a dihydroisoquinolinone bicyclic core. J Med Chem.

[38] Chacon Simon S, Wang F, Thomas LR, Phan J, Zhao B, Olejniczak ET (2020). Discovery of WD repeat-containing protein 5 (WDR5)-MYC inhibitors using fragment-based methods and structure-based design. J Med Chem.

[39] Borgenvik A, Cancer M, Hutter S, Swartling FJ (2020). Targeting MYCN in molecularly defined malignant brain tumors. Front Oncol.

[40] Weissmiller AM, Wang J, Lorey SL, Howard GC, Martinez E, Liu Q (2019). Inhibition of MYC by the SMARCB1 tumor suppressor. Nat Commun.

[41] Woodley CM, Romer AS, Wang J, Guarnaccia AD, Elion DL, Maxwell JN (2021). Multiple interactions of the oncoprotein transcription factor MYC with the SWI/SNF chromatin remodeler. Oncogene.

[42] Chen X, Xu H, Yuan P, Fang F, Huss M, Vega VB (2008). Integration of external signaling pathways with the core transcriptional network in embryonic stem cells. Cell.

[43] Martin M (2011). Cutadapt removes adapter sequences from high-throughput sequencing reads. EMBnet J.

[44] Langmead B, Trapnell C, Pop M, Salzberg SL (2009). Ultrafast and memory-efficient alignment of short DNA sequences to the human genome. Genome Biol.

[45] Feng J, Liu T, Qin B, Zhang Y, Liu XS (2012). Identifying ChIP-seq enrichment using MACS. Nat Protoc.

[46] Stark RB, G.D. DiffBind: differential binding analysis of ChIP-seq peak data. Bioconductor. 2011, 10.1038/s41388-021-01804-7.

[47] Bumpous L. Determining the impact of WDR5 on the ability of N-MYC to bind chromatin. Middle Tennessee State University; 2022.

