# Supplementary material for: WDR5 facilitates recruitment of N-MYC to conserved WDR5 gene targets in neuroblastoma cell lines
Source: Oncogenesis. 2023 Jun 19;12(1):32. doi: 10.1038/s41389-023-00477-z (PMC10279693; doi:10.1038/s41389-023-00477-z)
Supplement: Supplementary file 1 — Supplementary Material [file 41389_2023_477_MOESM1_ESM.pdf]

Supplementary Material

WDR5 facilitates recruitment of N-MYC to conserved WDR5 gene targets in neuroblastoma cell lines

Bumpous LA, Moe KC, et al.

Included figures:

Supplementary Figure 1. Characteristics of N-MYC and WDR5 chromatin binding in the N-MYC amplified neuroblastoma cell line, CHP-134.

Supplementary Figure 2. Characteristics of N-MYC and WDR5 chromatin binding in the N-MYC amplified neuroblastoma cell line, IMR-32.

Excel files:

Supplementary Table 1. Annotation of all N-MYC-WDR5 cobound sites in CHP-134 cells. ChIP-seq peaks were called for N-MYC and WDR5 separately and then peaks that overlapped were deemed as N-MYC-WDR5 cobound sites. Each N-MYC-WDR5 cobound site was annotated using Homer as described in materials and methods.

Supplementary Table 2. Annotation of all N-MYC-WDR5 cobound sites in IMR-32 cells. ChIP-seq peaks were called for N-MYC and WDR5 separately and then peaks that overlapped were deemed as N-MYC-WDR5 cobound sites. Each N-MYC-WDR5 cobound site was annotated using Homer as described in materials and methods.

Supplementary Table 3. Primer sequences. Primers used in this study for ChIP-QPCR results presented in Figure 1 and Figure 2.

Extended Materials and Methods

Cell culture and cell line engineering. CHP-134 and Kelly cells were obtained from Sigma. IMR-32, SHEP, SK-N-AS, and Be(2)C cell lines were used previously (1). HEK293T are in-house stocks. Neuroblastoma cell lines and various engineered derivatives are regularly tested to confirm absence of mycoplasma (MP Biomedicals) and integrity based on STR profiling (ATCC). All neuroblastoma cell lines were maintained in RPMI 1640 with l-glutamine (Corning) with 10% fetal bovine serum (FBS) and 1% Penicillin/Streptomycin while HEK293T cells were maintained in DMEM (Corning) with 10% FBS and 1% Penicillin/Streptomycin. CHP-134 cells expressing WDR5 that can be degraded via the dTAG approach (called "DTWDR5" in this study) were previously engineered (1). In order to generate lentiviral particles for engineering SHEP and SK-N-AS cell lines, HEK293T cells were transfected with the appropriate WT-N-MYC, WBM-N-MYC, or GFP vector (see "plasmid generation"), along with the psPAX2 and pMD2.G packing and envelope plasmids as previously described (2, 3). Viral supernatant was collected in normal SHEP/SK-N-AS maintenance media and used to transduce the cells. All cells were transduced for two days and allowed to recover for one day prior to selection. Following selection in 0.6 mg/ml G418 (Sigma), media on cells was replaced with normal maintenance media containing Tet-approved FBS (Clontech) instead of normal FBS.

Plasmid Generation. pFlag vectors were first created by PCR amplification of DNA sequences encoding N-MYC from the pTH-MYCN vector (4) with subsequent insertion into the multiple cloning site of the pFlag-C2 vector (5). Tet-inducible lentiviral vectors were then generated by PCR amplification of N-MYC encoding sequences from pFlag-N-MYC following insertion into the pENTR1A vector (6), along with sequences encoding a HA-epitope tag through Gibson assembly (New England BioLabs). Mutation of the WBM site of N-MYC was performed using whole-plasmid mutagenesis with primers that introduce three mutations within the WDR5 binding motif in Myc Box IIIb of N-MYC (aa: I277E/V279E/V280E). Gateway cloning (Invitrogen) was used to insert each fragment into the pInducer20 lentiviral acceptor vector (7). pInducer 20 vector containing EGFP DNA sequences was previously constructed (2) and used to generate the control SHEP cell line in this study. Fragments encoding amino acids 133-343 of WT-N-MYC were introduced into the pFlag-GALHA vector (5) following amplification out of the appropriate source plasmid and the WBM mutation introduced as described above. All plasmid sequences were confirmed by Sanger sequencing. pTH-MYCN was a gift from William A. Weiss (Addgene plasmid # 35416 ; http://n2t.net/addgene:35416 ; RRID:Addgene_35416). pENTR1A no ccDB (w48-1) was a gift from Eric Campeau & Paul Kaufman (Addgene plasmid # 17398 ; http://n2t.net/addgene:17398 ; RRID:Addgene_17398). pInducer20 was a gift from Stephen Elledge (Addgene plasmid # 44012 ; http://n2t.net/addgene:44012 ; RRID:Addgene_44012).

Protein lysates and immunoprecipitation experiments. Approximately 2.0 x 10^6 DTWDR5 CHP-134 cells were treated with either 500 nM dTAG47 or matched dimethyl sulfoxide (DMSO) control for 4 hr prior to protein lysate collection. For engineered SHEP and SK-N-AS

cells, protein lysates were generated from approximately 2.0 x 10^6 engineered cells induced with 1μg/ml doxycycline for 24 hr. Doxycycline was made fresh each week, sterile-filtered, and stored at 4°C prior to use. All cells were lysed by brief sonication in ice-cold lysis buffer (150 mM Tris-HCl pH 8.0, 5 mM EDTA, 150 mM NaCl, and 1% Triton X-100) supplemented with PMSF and protease inhibitor cocktail (Roche). Debris were cleared by centrifugation for 10 min at 13,000 RPM and the BioRad Bradford assay was used to determine protein concentrations and normalize samples to each other. For Flag-IP experiments, HEK293T cells were transiently transfected for 48 hr with indicated plasmids using the calcium phosphate transfection method. Cells were lysed as described for protein lysates. These lysates were normalized to each other and used as input for immunoprecipitation with M2(Flag)-conjugated agarose beads (Sigma) overnight at 4°C. M2-beads were blocked with 1% bovine serum albumin (BSA) for a minimum of 30 minutes in lysis buffer prior to being added to samples. For endogenous N-MYC-IP in CHP-134 cells, approximately 8.0 x 10^6 cells were treated with MG132 for 1.5 hours and then nuclei extracted in Buffer A (1 M HEPES pH 7.9, 0.5 M EDTA, 1 M KCl) with 0.4% NP-40. Nuclei were treated with lysis buffer and sonication as described above to obtain soluble proteins. Immunoprecipitation was performed with 5 μl of an antibody against N-MYC (Cell Signaling, 51705) or 800 ng of normal rabbit IgG control (Cell Signaling, 2729) overnight at 4°C. The following day, protein A agarose (Fisher Scientific) was blocked for 30 min with 1% BSA in lysis buffer and allowed to bind immunocomplexes for 2-4 hr at 4°C. Following binding of IP samples to either the (Flag)-conjugated agarose beads or protein A agarose, all IP samples were washed four times in ice-cold lysis buffer, transferred to a fresh tube, and then boiled in 2.5X SDS-loading dye. All protein lysates or IP inputs were prepared for Western blot analysis

by boiling in 1X SDS-loading dye. Endogenous N-MYC IPs were performed two independent times and transient transfection experiments were performed three independent times.

Western blot. All proteins were resolved by SDS-PAGE and transferred to PVDF membranes (PerkinElmer). Blots were blocked in 5% milk in 1X TBS (50 mM Tris, pH 7.6 and 150 mM NaCl) containing 0.1% Tween-20 (TBS-T). Each blot was probed with primary antibodies overnight at 4°C. The next day, blots were washed three times with TBS-T and secondary HRP-antibody was added at 1:5000 dilution to the appropriate blots for 1 hr at room temperature. Following three more washes in TBS-T, all blots were visualized using Clarity Western ECL or Max ECL substrate (Bio-Rad) on a ChemiDoc MP (Bio-Rad) instrument. Antibodies used for Western blot in this study include: Flag-HRP (Cell Signaling, 86861), WDR5 (Cell Signaling, 13105), MAX (Cell Signaling, 47339), GFP (Cell Signaling, 2956), N-MYC (Cell Signaling, 51705), and GAPDH-HRP (Cell Signaling, 8884).

References for Extended Materials and Methods

1. Bryan AF, Wang J, Howard GC, Guarnaccia AD, Woodley CM, Aho ER, et al. WDR5 is a conserved regulator of protein synthesis gene expression. Nucleic Acids Res. 2020.
2. Weissmiller AM, Wang J, Lorey SL, Howard GC, Martinez E, Liu Q, et al. Inhibition of MYC by the SMARCB1 tumor suppressor. Nat Commun. 2019;10(1):2014.
3. Woodley CM, Romer AS, Wang J, Guarnaccia AD, Elion DL, Maxwell JN, et al. Multiple interactions of the oncoprotein transcription factor MYC with the SWI/SNF chromatin remodeler. Oncogene. 2021;40(20):3593-609.
4. Weiss WA, Aldape K, Mohapatra G, Feuerstein BG, Bishop JM. Targeted expression of MYCN causes neuroblastoma in transgenic mice. Embo J. 1997;16(11):2985-95.
5. Thomas LR, Wang Q, Grieb BC, Phan J, Foshage AM, Sun Q, et al. Interaction with WDR5 promotes target gene recognition and tumorigenesis by MYC. Mol Cell. 2015;58(3):440-52.
6. Campeau E, Ruhl VE, Rodier F, Smith CL, Rahmberg BL, Fuss JO, et al. A versatile viral system for expression and depletion of proteins in mammalian cells. PLoS One. 2009;4(8):e6529.

7. Meerbrey KL, Hu G, Kessler JD, Roarty K, Li MZ, Fang JE, et al. The pINDUCER lentiviral toolkit for inducible RNA interference in vitro and in vivo. Proc Natl Acad Sci U S A. 2011;108(9):3665-70.

Supplementary Figure 1

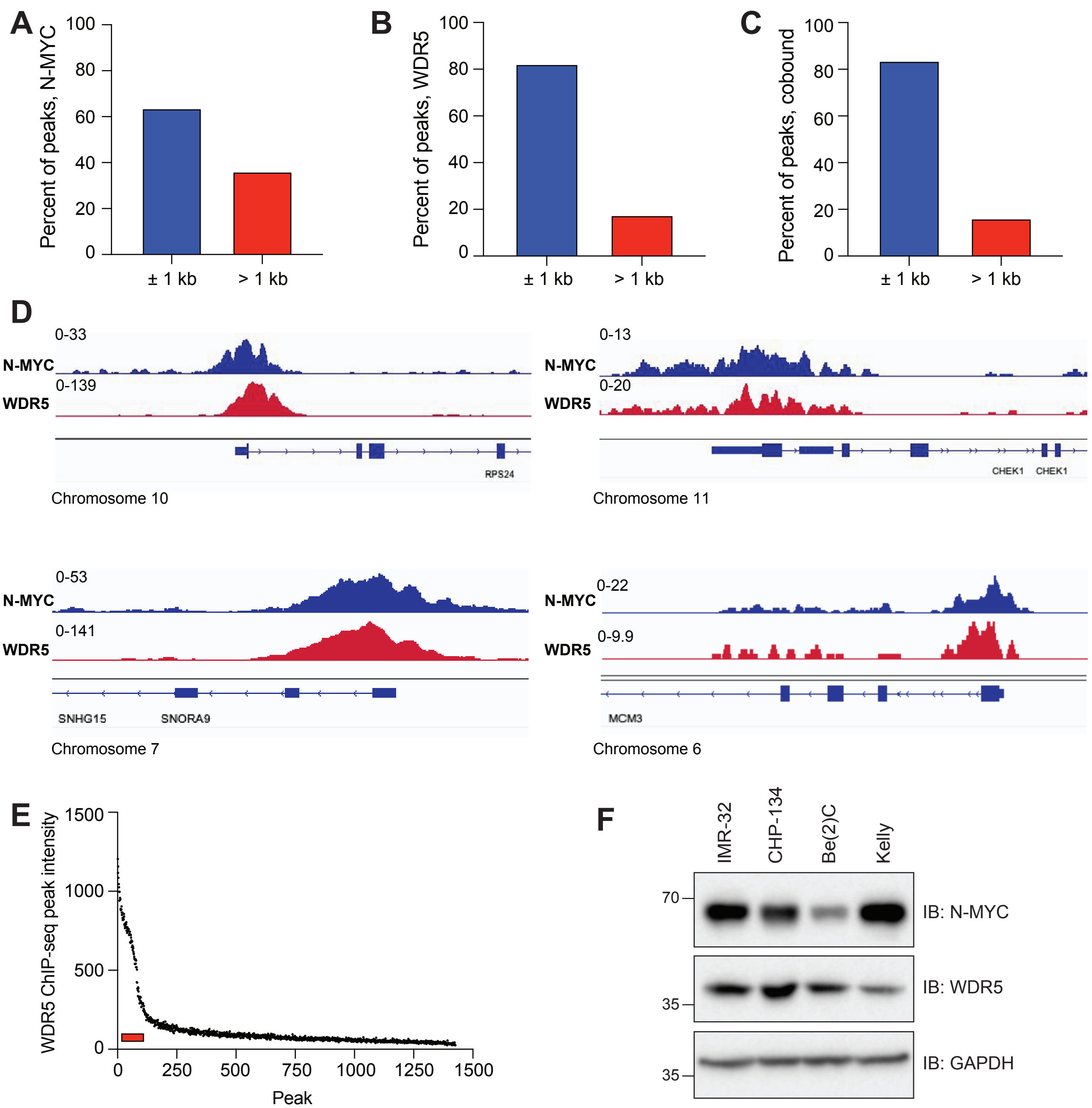

Supplementary Figure 1. Characteristics of N-MYC and WDR5 chromatin binding in the N-MYC amplified neuroblastoma cell line, CHP-134. (**A**) Percent of N-MYC ChIP-seq peaks that fall within 1 kb of a transcription start site (TSS) based on annotation of peaks called. Analysis was also performed for WDR5 ChIP-seq peaks (**B**) and N-MYC-WDR5 overlapped peaks (**C**). (**D**) IGV screenshot examples of normalized ChIP-seq peaks at sites called as N-MYC-WDR5 cobound. Left two genes (*RPS24* and *SNHG15*) are among the context-independent, conserved WDR5-bound sites identified in previous studies and right two genes (*CHEK1* and *MCM3*) are non-conserved WDR5-bound sites. (**E**) ChIP-seq peaks for WDR5 ranked in order of intensity. Red rectangular bar within the graph indicates the WDR5 ChIP-seq peak intensity at conserved WDR5-bound sites. (F) Western blot was used to compare N-MYC and WDR5 protein levels in indicated cell lines.

Supplementary Figure 2

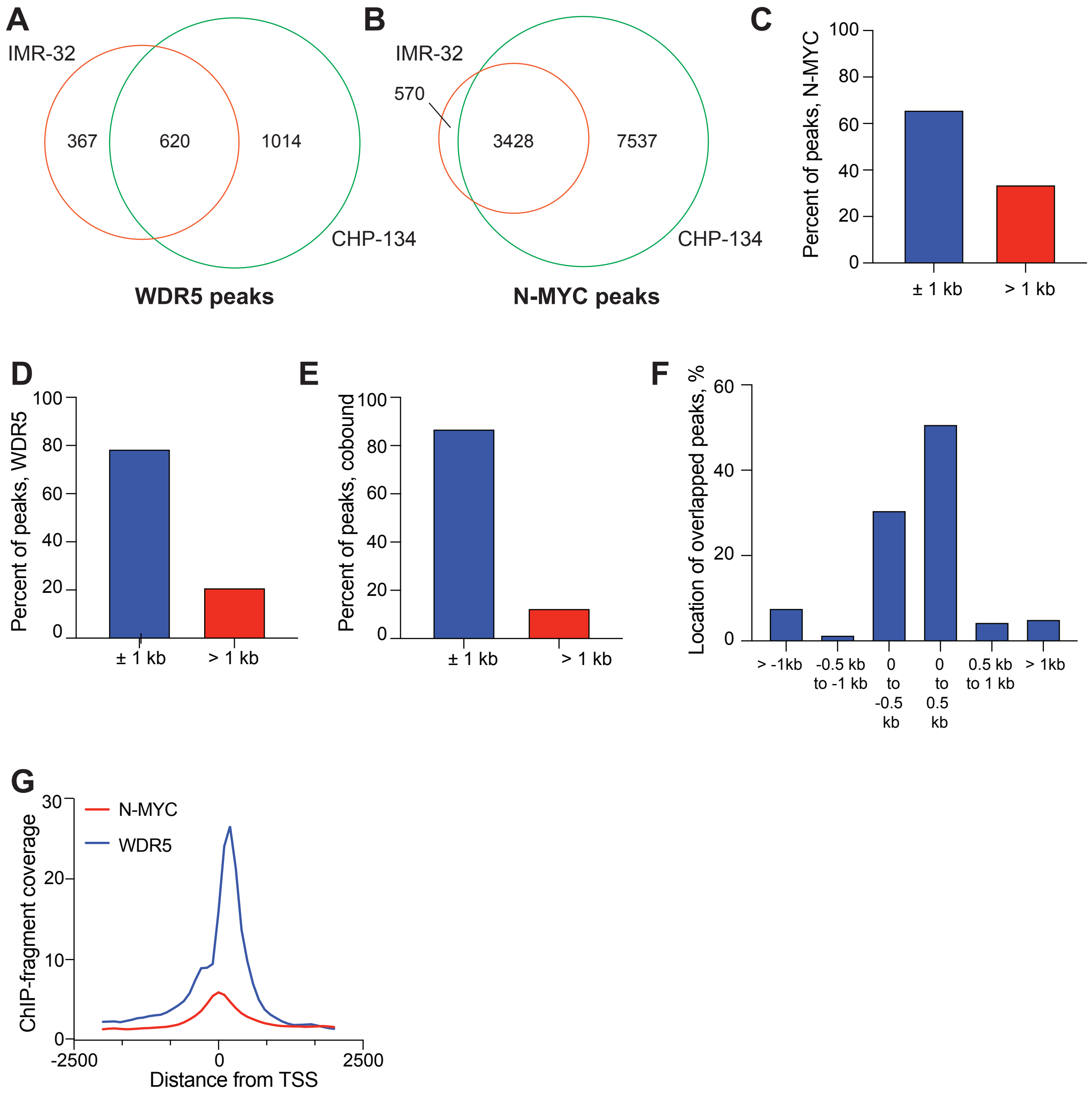

Supplementary Figure 2. Characteristics of N-MYC and WDR5 chromatin binding in the N-MYC amplified neuroblastoma cell line, IMR-32. (A) Venn diagram showing the overlap between WDR5 peaks detected in IMR-32 and CHP-134 cells. (B) Venn diagram showing the overlap between N-MYC peaks detected in IMR-32 and CHP-134 cells (C) Percent of N-MYC ChIP-seq peaks that fall within 1 kb of a TSS based on annotation of peaks. (D) Percent of WDR5 ChIP-seq peaks that fall within 1 kb of a TSS based on annotation of peaks. (E) Percent of N-MYC-WDR5 overlapped peaks that fall within 1 kb of a TSS based on annotation of peaks. (F) Distribution of N-MYC-WDR5 cobound sites based on annotation of overlapped peaks to their nearest TSS. (G) Average normalized ChIP-seq fragment coverage for N-MYC (red) and WDR5 (blue) at all N-MYC-WDR5 cobound sites.
